# Dicationic Herbicidal Ionic Liquids Comprising Two Active Ingredients Exhibiting Different Modes of Action

**DOI:** 10.1021/acs.jafc.1c07750

**Published:** 2022-02-16

**Authors:** Juliusz Pernak, Michał Niemczak, Tomasz Rzemieniecki, Katarzyna Marcinkowska, Tadeusz Praczyk

**Affiliations:** † Department of Chemical Technology, Poznan University of Technology, Poznan 60-965, Poland; ‡ Institute of Plant Protection - National Research Institute, Poznan 60-318, Poland

**Keywords:** HILs, bis(ammonium), herbicide, sulfonylurea, phenoxy acids, weed resistance

## Abstract

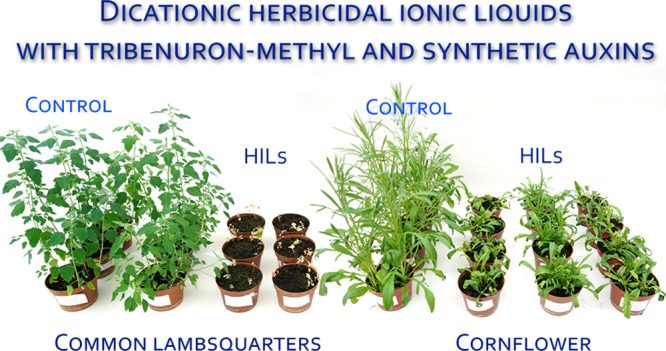

In the framework of this study, dicationic herbicidal ionic liquids (HILs) containing tetramethylene-1,4-bis(decyldimethylammonium) and dodecylmethylene-1,12-bis(decyldimethylammonium), including two different herbicidal anions exhibiting different modes of action, were synthesized and characterized. One herbicide incorporated into the HILs was a tribenuron-methyl belonging to ALS inhibitors, while the second herbicidal anion was a synthetic auxin that acts as a growth regulator, namely 2,4-dichlorophenoxyacetate (2,4-D), 2-(2,4-dichlorophenoxy)propionate, (2,4-DP), 2,4,5-trichlorophenoxyacetate (2,4,5-T), 4-chloro-2-methylphenoxyacetiate (MCPA), 2-(4-chloro-2-methylphenoxy)propionate (MCPP), and 4-chlorophenoxyacetate (4-CPA). The obtained products were found to be unstable and decomposed, which can be attributed to the presence of an additional methyl group within the sulfonylurea bridge of the tribenuron-methyl. The synthesized HILs exhibited good affinity with polar and semipolar solvents, with ethyl acetate and hexane as the only solvents that did not dissolve the HILs. Greenhouse tests demonstrated that most of the obtained HILs were more effective than the reference herbicide containing tribenuron-methyl. The length of the alkyl chain in the cation also influenced the effectiveness of the HILs. Better effects were observed for dodecylmethylene-1,12-bis(decyldimethylammonium) cations compared to tetramethylene-1,4-bis(decyldimethylammonium). Therefore, the novel dicatonic HILs showed to integrate the advent of the combination of the different herbicides into a single molecule, enhance herbicidal efficacy, and reduce the risk of weed resistance due to the various modes of action of the applied treatment.

## Introduction

The use of herbicides has been linked to the profitability of agricultural production; however, it should be noted that only 5% of the applied active ingredients affects unwanted vegetation.^1^ The rest of the applied pesticide contributes to environmental loading and may accumulate in the soil,^2^ leach into groundwater,^3,4^ or volatilize into the atmosphere.^5,6^ Moreover, although currently approved pesticides are expected to be readily biodegradable, the degraded substances can be harmful to human and animal health.^7^ It is therefore necessary to actively search for solutions to help reduce the scale of these problems. The conversion of known herbicides into ionic organic salts, which results in herbicidal ionic liquids (HILs), is a promising strategy to offset the significant drawbacks of currently known herbicides.^8−10^ A particularly effective approach is to combine herbicidal anions with cations that provide surface activity to the resulting compounds. This allows the HIL activity to be significantly increased compared to the initial substance without the use of adjuvants, resulting in the reduction of pesticide usage.^9^ This concept was further developed and indicated that it is possible to combine two herbicidal anions into one ionic system in the form of double salt herbicidal ionic liquids (DSHILs).^11^ Further studies have shown that there is a potential synergy of action between two or three herbicidally active anions in DSHILs, resulting in further enhancement of biological activity compared to HILs with a single anion.^12,13^

Gemini surfactants are a group of surface-active chemical compounds with surface activities significantly higher than those of classical surfactants.^14^ For example, 1,6-hexamethylene-bis(*N*-hydroxyethyl-*N*-methyl-*N*-octadecylammonium) dibromide has a critical micellization concentration of 0.046 ± 0.004 mmol L^–1^,^15^ almost 180 times lower than sodium dodecyl sulfate.^16^ Due to their very high surface activity, they can act as corrosion inhibitors^17,18^ or as elements of innovative compounds with biological activity, e.g., insect antifeedants.^19^ There have also been successful attempts to synthesize new salts with bis(ammonium) cations and herbicidal anions.^20^ The biological activity of bis(ammonium) salts can be effectively tuned by choosing an appropriate length of both the alkyl substituents and alkylene spacer.^21,22^

Tribenuron-methyl is a sulfonylurea herbicide capable of controlling the growth of dicotyledonous weeds even when applied at a dose equal to 15 g ha^–1^. According to the application risk assessment published by EFSA,^23^ this active ingredient exhibits low mammalian toxicity and limited environmental impact. EFSA assessment also states that it is very unlikely for tribenuron-methyl to be carcinogenic toward humans. For optimal biological activity, this compound must be applied in solutions with surface-active adjuvants. However, it should be noted that there is a high risk of acquisition of resistance to tribenuron-methyl,^24,25^ so it is recommended for use in mixtures with other herbicides or to apply herbicides with a different mechanism of action in subsequent agronomic treatments. Ready-made formulations containing tribenuron-methyl and another herbicide are also available, for example, Granstar Power 74.4 SG (FMC Agro Polska), which also contains the compound mecoprop-p, a member of the phenoxy acid group. Such formulations are characterized by a broader spectrum of action than herbicides containing tribenuron-methyl alone.

To create a more effective formulation, the transformation of tribenuron-methyl to the IL form was first proposed in this study. To further improve the sulfonylurea activity, phenoxy acid anions were also introduced into the newly developed compounds, similar to that of standard commercially available mixtures. In addition, to make the use of adjuvants in the preparation of the spray mixture unnecessary, two highly surface-active bis(ammonium) cations with varying alkylene linker lengths were used as counterions. The effect of the structure of the amphiphilic cation and the anion derived from phenoxy acid on the physicochemical properties of the resulting products, as well as biological activity against undesirable plants, was also determined.

## Materials

2-(2,4-Dichlorophenoxy)propanoic acid (2,4-DP) (purity 97%), (2,4-dichlorophenoxy)acetic (2,4-D) (purity 97%), 3,6-dichloro-2-methoxybenzoic acid (dicamba) (98%), 2-(4-chloro-2-methylphenoxy)propanoic acid (MCPP-P) (purity 97%), (4-chloro-2-methylphenoxy)acetic acid (MCPA) (purity 97%) and tribenuron-methyl (TBM) (purity 97%) were obtained from PESTINOVA (Jaworzno, Poland). 4-Chlorophenoxyacetatic acid (4-CPA) (purity 98%) and 2,4,5-trichlorophenoxyacetic acid (2,4,5-T) (purity 95%) were purchased from Sigma-Aldrich (Poznan, Poland). Sodium hydroxide (purity 99%, potassium hydroxide (purity 85%) and all solvents (methanol, acetonitrile, acetone, hexane, toluene, chloroform, isopropanol, DMSO, and ethyl acetate) were purchased from Avantor (Gliwice, Poland). Deionized water with a conductivity of <0.1 μS cm^–1^ from demineralizer HLP Smart 1000 (Hydrolab, Poland) was used for solubility and surface activity measurement. All reagents and solvents were used without further purification.

## Synthesis

### Preparation of Dicationic Bromides

The appropriate dibromoalkane (0.1 mol) was poured into a round-bottom flask. Next, decyldimethylamine with a 10% excess and 100 mL of acetonitrile were added. The synthesis was conducted at the boiling point temperature of the solvent for 24 h. The solvent was then removed by a vacuum evaporator, and 100 mL of ethyl acetate was added. The product, which precipitated in the form of a white solid, was isolated by filtration, washed with small portions of ethyl acetate (5 × 10 mL), and dried under reduced pressure at 65 °C for 24 h.

### Synthesis of Dicationic HILs

The appropriate dicationic bromide (0.1 mol) was dissolved in 50 mL of anhydrous methanol, followed by the addition of a mixture consisting of 0.1 mol of sodium salt of tribenuron-methyl (sulfonylurea, TB-M) and 0.1 mol of potassium salt of selected synthetic auxin (3,6-dichloro-2-methoxybenzoate (dicamba) or phenoxy acid from the group: 2,4-dichlorophenoxyacetate (2,4-D), 2-(2,4-dichlorophenoxy)propionate, (2,4-DP), 2,4,5-trichlorophenoxyacetate (2,4,5-T), 4-chloro-2-methylphenoxyacetiate (MCPA), 2-(4-chloro-2-methylphenoxy)propionate (MCPP), and 4-chlorophenoxyacetate (4-CPA)). The reaction was conducted for 60 min, and the precipitated inorganic byproduct (sodium/potassium bromide) was removed by filtration. Afterward, the residue was dissolved in 50 mL of acetone, and traces of inorganic salts and other impurities were filtered off. After the evaporation of acetone, the compounds were dried under vacuum for 24 h at 65 °C.

#### Tetramethylene-1,4-Bis(decyldimethylammonium) Tribenuron-methyl, 2,4-Dichlorophenoxyacetate (1:1) (**1**)

^1^H NMR (DMSO-*d*_6_) δ [ppm]: 0.88 (t, *J* = 6.8 Hz, 6H), 1.16–1.35 (m, 28H), 1.65–1.77 (m, 4H) 1.98–2.06 (m, 4H), 2.34 (s, 3H), 3.08 (s, 12H), 3.23–3.38 (m, 4H), 3.51 (s, 3H), 3.67–3.82 (m, 4H), 3.79–3.93 (m, 6H), 4.21 (s, 2H), 6.73 (d, *J* = 8.1 Hz, 1H), 6.93–7.01 (m, 2H), 7.36–7.49 (m, 3H), 8.14 (d, *J* = 8.2 Hz, 1H).

^13^C NMR (DMSO-*d*_6_) δ [ppm]: 177.18, 172.57, 170.62, 168.69, 166.45, 158.78, 156.03, 143.19, 131.70, 129.52, 128.84, 128.66, 127.58, 126.56, 122.51, 119.02, 113.13, 67.69, 63.07, 62.02, 54.10, 52.16, 49.88, 34.22, 31.38, 29.03, 28.68, 28.61, 25.89, 25.30, 22.12, 21.84, 19.06, 18.97, 14.01.

#### Tetramethylene-1,4-bis(decyldimethylammonium) Tribenuron-methyl, (*RS*)-2-(2,4-Dichlorophenoxy)propionate (1:1) (**2**)

^1^H NMR (DMSO-*d*_6_) δ [ppm]: 0.86 (t, *J* = 6.7 Hz, 6H), 1.16–1.34 (m, 28H), 1.42 (d, *J* = 6.7 Hz, 3H), 1.57–1.77 (m, 8H), 2.32 (s, 3H), 3.03 (s, 12H), 3.17 (s, 3H), 3.22–3.30 (m, 4H), 3.32–3.41 (m, 4H), 3.76 (s, 3H), 3.84 (s, 3H), 4.32 (q, *J* = 6.7 Hz, 1H), 6.86 (d, *J* = 8.9 Hz, 1H), 7.25 (dd, *J*_12_ = 2.6 Hz, *J*_13_ = 9.0 Hz, 1H), 7.37–7.41 (m, 1H), 7.44 (d, *J* = 2.6 Hz, 1H), 7.48–7.56 (m, 2H), 8.04–8.10 (m, 1H).

^13^C NMR (DMSO-*d*_6_) δ [ppm]: 177.65, 172.66, 170.17, 168.80, 166.87, 157.83, 153.32, 142.60, 131.86, 129.97, 129.02, 128.69, 127.50, 127.08, 122.73, 121.75, 115.42, 76.48, 63.21, 62.04, 54.12, 52.27, 49.86, 34.14, 31.33, 29.02, 28.66, 28.57, 25.93, 25.25, 22.14, 21.79, 19.12, 19.01, 13.98.

#### Tetramethylene-1,4-bis(decyldimethylammonium) Tribenuron-methyl, 2,4,5-Trichlorophenoxyacetate (1:1) (**3**)

^1^H NMR (DMSO-*d*_6_) δ [ppm]: 0.88 (t, *J* = 6.9 Hz, 6H), 1.17–1.36 (m, 28H), 1.62–1.74 (m, 4H) 2.00–2.10 (m, 4H), 2.38–2.48 (m, 3H), 3.15 (s, 12H), 3.27–3.35 (m, 4H), 3.42 (s, 3H), 3.69–3.77 (m, 4H), 3.84–3.99 (m, 6H), 7.29 (d, *J* = 11.1 Hz, 1H), 7.34–7.63 (m, 4H), 8.03–8.22 (m, 2H).

^13^C NMR (DMSO-*d*_6_) δ [ppm]: 177.45, 170.49, 169.41, 167.18, 159.03, 142.33, 131.77, 130.40, 129.81, 129.14, 128.56, 127.69, 127.13, 123.02, 119.45, 71.87, 65.12, 63.58, 54.53, 52.66, 50.42, 34.53, 31.67, 29.41, 29.33, 29.12, 26.10, 25.47, 22.55, 22.46, 19.38, 14.03.

#### Tetramethylene-1,4-bis(decyldimethylammonium) Tribenuron-methyl, 4-Chloro-2-methylphenoxyacetate (1:1) (**4**)

^1^H NMR (CDCl_3_) δ [ppm]: 0.88 (t, *J* = 6.8 Hz, 6H), 1.12–1.36 (m, 28H), 1.46 (d, *J* = 6.6 Hz, 3H), 1.49–1.62 (m, 4H), 1.80–1.83 (m, 4H), 2.36 (s, 3H), 3.06 (s, 12H), 3.12–3.23 (m, 4H), 3.42 (s, 3H), 3.45–3.56 (m, 4H), 3.81 (s, 3H), 3.83 (s, 3H), 4.25 (s, 2H), 6.69 (d, *J* = 8.2 Hz, 1H), 6.93–7.05 (m, 2H), 7.38–7.53 (m, 3H), 8.17 (d, *J* = 8.5 Hz, 1H).

^13^C NMR (CDCl_3_) δ [ppm]: 177.48, 170.40, 169.26, 167.34, 159.05, 155.38, 142.25, 131.80, 130.42, 129.86, 128.91, 128.57, 127.59, 126.32, 123.87, 113.42, 76.03, 64.85, 63.63, 54.51, 52.69, 50.30, 34.52, 31.66, 29.42, 29.18, 29.12, 26.15, 25.69, 22.63, 19.38, 16.21, 14.03.

#### Tetramethylene-1,4-bis(decyldimethylammonium) Tribenuron-methyl, (*RS*)-2-(4-Chloro-2-methylphenoxy)propionate (1:1) (**5**)

^1^H NMR (CDCl_3_) δ [ppm]: 0.88 (t, *J* = 6.9 Hz, 6H), 1.11–1.34 (m, 28H), 1.47 (d, *J* = 6.5 Hz, 3H), 1.50–1.60 (m, 4H), 1.79–1.81 (m, 4H), 2.40 (s, 3H), 2.97 (s, 12H), 3.06–3.14 (m, 4H), 3.39 (s, 3H), 3.41–3.54 (m, 4H), 3.84 (s, 3H), 3.88 (s, 3H), 4.30–4.43 (m, 1H), 6.69 (d, *J* = 8.3 Hz, 1H), 6.95–7.02 (m, 2H), 7.38–7.51 (m, 3H), 8.16 (d, *J* = 8.5 Hz, 1H).

^13^C NMR (CDCl_3_) δ [ppm]: 177.58, 170.53, 169.31, 167.28, 159.04, 155.71, 141.79, 131.65, 130.42, 129.87, 128.92, 128.70, 127.84, 126.21, 123.87, 113.46, 76.08, 64.66, 63.62, 54.48, 52.70, 50.33, 34.47, 31.83, 29.37, 29.24, 29.12, 26.18, 25.46, 22.59, 19.32, 16.30, 14.02.

#### Tetramethylene-1,4-bis(decyldimethylammonium) Tribenuron-methyl, 4-Chlorophenoxyacetate (1:1) (**6**)

^1^H NMR (CDCl_3_) δ [ppm]: 0.87 (t, *J* = 6.9 Hz, 6H), 1.14–1.40 (m, 28H), 1.53–1.79 (m, 8H), 2.35 (s, 3H), 3.11 (s, 12H), 3.14–3.28 (m, 4H), 3.37 (s, 3H), 3.42–3.54 (m, 4H), 3.85 (s, 3H), 3.87 (s, 3H), 4.25 (s, 2H), 6.93–7.07 (m, 4H), 7.38–7.53 (m, 3H), 8.17 (d, *J* = 8.4 Hz, 1H).

^13^C NMR (CDCl_3_) δ [ppm]: 177.38, 170.26, 168.44, 167.10, 159.15, 142.37, 131.92, 131.01, 130.30, 129.76, 128.97, 128.53, 123.05, 119.54, 72.27, 65.09, 63.62, 54.47, 52.39, 50.41, 34.48, 31.66, 29.40, 29.27, 29.14, 26.08, 25.52, 22.36, 22.48, 19.40, 14.03.

#### Tetramethylene-1,4-bis(decyldimethylammonium) Tribenuron-methyl, 3,6-Chloro-2-methoxybenzoate (1:1) (**7**)

^1^H NMR (DMSO-*d*_6_) δ [ppm]: 0.86 (t, *J* = 6.8 Hz, 6H), 1.18–1.33 (m, 28H), 1.57–1.84 (m, 8H), 2.31 (s, 3H), 3.03 (s, 12H), 3.21–3.32 (m, 7H), 3.33–3.41 (m, 4H), 3.78 (s, 3H), 3.81 (s, 3H), 3.84 (s, 3H), 7.07 (d, *J* = 8.5 Hz, 1H), 7.21 (d, *J* = 8.5 Hz, 1H), 7.39–7.54 (m, 3H), 8.23 (d, *J* = 8.5 Hz, 1H).

^13^C NMR (DMSO-*d*_6_) δ [ppm]: 177.04, 172.07, 170.12, 168.86, 167.18, 158.93, 151.15, 143.11, 131.77, 129.50, 129.03, 128.68, 127.51, 126.87, 125.31, 122.54, 119.12, 63.17, 61.97, 61.01, 53.68, 52.23, 50.91, 49.85, 34.40, 31.32, 29.04, 28.88, 28.67, 28.62, 25.80, 24.76, 22.12, 21.84, 18.97, 14.01.

#### Dodecylmethylene-1,12-Bis(decyldimethylammonium) Tribenuron-methyl, 2,4-Dichlorophenoxyacetate (1:1) (**8**)

^1^H NMR (DMSO-*d*_6_) δ [ppm]: 0.85 (t, *J* = 6.9 Hz, 6H), 1.16–1.37 (m, 44H), 1.54–1.70 (m, 8H), 2.14 (s, 3H), 2.40 (s, 3H), 3.01 (s, 12H), 3.19–3.26 (m, 8H), 3.30 (s, 3H), 3.78 (s, 3H), 3.83 (s, 3H), 4.19 (s, 2H), 6.68 (d, *J* = 8.6 Hz, 1H), 7.07 (dd, *J*_12_ = 2.6 Hz, *J*_13_ = 8.8 Hz, 1H), 7.12 (d, *J* = 2.9 Hz, 1H), 7.38–7.52 (m, 3H), 8.19 (d, *J* = 8.6 Hz, 1H).

^13^C NMR (DMSO-*d*_6_) δ [ppm]: 176.99, 172.82, 170.56, 168.83, 166.87, 158.94, 156.00, 143.23, 131.75, 129.63, 128.98, 128.71, 127.76, 126.72, 122.60, 119.01, 112.87, 67.92, 62.76, 53.74, 52.01, 50.79, 49.93, 34.21, 31.32, 29.04, 28.87, 28.79, 28.71, 28.58, 28.52, 28.40, 26.86, 25.82, 25.15, 22.10, 21.67, 21.61, 15.98, 13.94.

#### Dodecylmethylene-1,12-bis(decyldimethylammonium) Tribenuron-methyl, (*RS*)-2-(2,4-Dichlorophenoxy)propionate (1:1) (**9**)

^1^H NMR (CDCl_3_) δ [ppm]: 0.88 (t, *J* = 6.9 Hz, 6H), 1.13–1.38 (m, 44H), 1.50 (d, *J* = 6.7 Hz, 3H), 1.54–1.70 (m, 8H), 2.40 (s, 3H), 3.14 (s, 12H), 3.21–3.33 (m, 8H), 3.40 (s, 3H), 3.86 (s, 3H), 3.89 (s, 3H), 4.43 (q, *J* = 6.7 Hz, 1H), 6.86 (d, *J* = 8.9 Hz, 1H), 7.09 (dd, *J_12_* = 2.6 Hz, *J_13_* = 8.9 Hz, 1H), 7.22 (d, *J* = 2.6 Hz, 1H), 7.37–7.52 (m, 3H), 7.48–7.56 (m, 2H), 8.15–8.22 (m, 1H).

^13^C NMR (CDCl_3_) δ [ppm]: 177.37, 176.71, 170.42, 169.25, 167.11, 152.89, 141.80, 131.63, 130.21, 129.67, 129.03, 128.86, 127.57, 124.54, 122.56, 115.48, 76.70, 63.52, 63.47, 54.34, 52.65, 50.91, 34.38, 31.56, 29.17, 29.04, 28.92, 28.79, 28.72, 26.02, 25.90, 25.31, 22.38, 18.93, 13.97.

#### Dodecylmethylene-1,12-bis(decyldimethylammonium) Tribenuron-methyl, 2,4,5-Trichlorophenoxyacetate (1:1) (**10**)

^1^H NMR (DMSO-*d*_6_) δ [ppm]: 0.86 (t, *J* = 6.8 Hz, 6H), 1.17–1.42 (m, 44H), 1.52–1.76 (m, 8H), 2.33 (s, 3H), 3.05 (s, 12H), 3.14 (s, 3H), 3.23–3.33 (m, 8H), 3.76 (s, 3H), 3.84 (s, 3H), 4.10 (s, 2H), 7.30 (d, *J* = 11.1 Hz, 1H), 7.32–7.64 (m, 4H), 8.03–8.25 (m, 2H).

^13^C NMR (DMSO-*d*_6_) δ [ppm]: 177.58, 172.66, 170.21, 168.80, 166.83, 157.79, 153.21, 142.82, 131.87, 130.05, 129.03, 128.69, 127.53, 127.20, 122.65, 121.67, 115.43, 76.51, 63.18, 62.04, 53.58, 52.01, 50.65, 49.93, 34.06, 31.32, 29.01, 28.87, 28.76, 28.70, 28.62, 28.45, 26.90, 25.74, 25.18, 22.09, 21.68, 21.62, 16.12, 13.94.

#### Dodecylmethylene-1,12-bis(decyldimethylammonium) Tribenuron-methyl, 4-Chloro-2-methylphenoxyacetate (1:1) (**11**)

^1^H NMR (CDCl_3_) δ [ppm]: 0.87 (t, *J* = 6.8 Hz, 6H), 1.13–1.46 (m, 44H), 1.42 (d, *J* = 6.6 Hz, 3H), 1.49–1.64 (m, 4H), 1.81–1.85 (m, 4H), 2.39 (s, 3H), 3.12 (s, 12H), 3.15–3.34 (m, 8H), 3.43 (s, 3H), 3.82 (s, 3H), 3.84 (s, 3H), 4.23 (s, 2H), 6.69 (d, *J* = 8.2 Hz, 1H), 6.92–7.07 (m, 2H), 7.34–7.51 (m, 3H), 8.16 (d, *J* = 8.5 Hz, 1H).

^13^C NMR (CDCl_3_) δ [ppm]: 177.58, 170.41, 169.34, 167.30, 158.97, 155.39, 142.18, 131.84, 130.25, 129.87, 128.85, 128.51, 127.63, 126.30, 123.94, 113.26, 76.13, 64.82, 63.46, 54.18, 52.41, 50.90, 34.42, 31.48, 29.21, 29.03, 28.86, 28.81, 28.73, 28.50, 26.09, 25.85, 25.31, 22.43, 18.91, 16.20, 14.01.

#### Dodecylmethylene-1,12-bis(decyldimethylammonium) Tribenuron-methyl, (*RS*)-2-(4-Chloro-2-methylphenoxy)propionate (1:1) (**12**)

^1^H NMR (CDCl_3_) δ [ppm] = 0.87 (t, *J* = 6.9 Hz, 6H), 1.12–1.38 (m, 44H), 1.46 (d, *J* = 6.5 Hz, 3H), 1.50–1.62 (m, 4H), 1.78–1.82 (m, 4H), 2.37 (s, 3H), 3.02 (s, 12H), 3.08–3.16 (m, 4H), 3.34 (s, 3H), 3.42–3.51 (m, 4H), 3.81 (s, 3H), 3.85 (s, 3H), 4.34 (q, *J* = 6.6 Hz, 1H), 6.68 (d, *J* = 8.2 Hz, 1H), 6.94–7.02 (m, 2H), 7.38–7.53 (m, 3H), 8.15 (d, *J* = 8.5 Hz, 1H).

^13^C NMR (CDCl_3_) δ [ppm] = 177.47, 170.50, 169.31, 167.24, 158.97, 155.55, 141.81, 131.57, 130.53, 129.89, 128.80, 128.68, 127.83, 125.90, 123.76, 112.92, 76.04, 64.66, 63.48, 54.49, 52.26, 50.93, 34.29, 31.47, 29.21, 29.10, 29.02, 28.87, 28.79, 28.72, 28.41, 26.14, 25.80, 25.29, 22.40, 18.96, 16.07, 14.06.

#### Dodecylmethylene-1,12-bis(decyldimethylammonium) Tribenuron-methyl, 4-Chlorophenoxyacetate (1:1) (**13**)

^1^H NMR (CDCl_3_) δ [ppm]: 0.87 (t, *J* = 6.9 Hz, 6H), 1.13–1.46 (m, 44H), 1.53–1.80 (m, 8H), 2.32 (s, 3H), 3.09 (s, 12H), 3.12–3.26 (m, 4H), 3.41 (s, 3H), 3.43–3.57 (m, 4H), 3.82 (s, 3H), 3.86 (s, 3H), 4.24 (s, 2H), 6.93–7.09 (m, 4H), 7.31–7.48 (m, 3H), 8.17 (d, *J* = 8.4 Hz, 1H).

^13^C NMR (CDCl_3_) δ [ppm]: 177.42, 170.20, 168.37, 167.18, 159.15, 142.34, 131.86, 131.02, 130.08, 129.86, 129.04, 128.47, 123.00, 119.25, 72.13, 65.21, 63.64, 54.38, 52.39, 50.66, 34.31, 31.60, 29.18, 29.13, 28.96, 28.92, 28.83, 28.67, 28.52, 26.11, 25.66, 25.29, 22.40, 18.99, 14.07.

#### Dodecylmethylene-1,12-bis(decyldimethylammonium) Tribenuron-methyl, 3,6-Chloro-2-methoxybenzoate (1:1) (**14**)

^1^H NMR (DMSO-*d*_6_) δ [ppm]: 0.87 (t, *J* = 6.8 Hz, 6H), 1.15–1.45 (m, 44H), 1.56–1.83 (m, 8H), 2.33 (s, 3H), 3.11 (s, 12H), 3.20–3.34 (m, 7H), 3.36–3.43 (m, 4H), 3.81 (s, 3H), 3.83 (s, 3H), 3.85 (s, 3H), 7.06 (d, *J* = 8.5 Hz, 1H), 7.21 (d, *J* = 8.5 Hz, 1H), 7.40–7.56 (m, 3H), 8.18 (d, *J* = 8.5 Hz, 1H).

^13^C NMR (DMSO-*d*_6_) δ [ppm]: 177.12, 172.08, 170.06, 168.83, 167.21, 155.11, 151.06, 143.04, 131.75, 129.52, 129.20, 128.74, 127.41, 126.93, 125.17, 122.51, 119.02, 63.07, 61.86, 61.03, 53.70, 52.15, 50.71, 34.15, 31.48, 29.23, 29.11, 29.02, 28.87, 28.81, 28.73, 28.40, 26.11, 25.56, 25.18, 22.32, 19.09, 14.02.

#### Tetramethylene-1,4-bis(decyldimethylammonium) Tribenuron-methyl, (*RS*)-2-(4-Chloro-2-methylphenoxy)propionate (1:1) (**5**), after Decomposition

^1^H NMR (DMSO-*d*_6_) δ [ppm]: 0.86 (t, *J* = 6.9 Hz, 6H), 1.18–1.34 (m, 28H), 1.43 (d, *J* = 6.6 Hz, 3H), 1.59–1.76 (m, 8H), 2.21 (s, 1.2H), 2.26 (s, 0.8H), 2.76–2.82 (m, 2H) 3.03 (s, 12H), 3.17 (s, 1.6H), 3.22–3.29 (m, 4H), 3.30 (s, 1.4H), 3.33–3.41 (m, 4H), 3.74 (s, 3H), 3.80 (s, 1H), 3.84 (s, 2H), 4.40 (q, *J* = 6.7 Hz, 1H), 6.87 (d, *J* = 9.0 Hz, 1H), 7.25 (dd, *J*_12_ = 2.6 Hz, *J*_13_ = 9.0 Hz, 1H), 7.31–7.35 (m, 1H), 7.43–7.53 (m, 3H), 7.57–7.64 (m, 0.4H), 7.64–7.69 (m, 0.2H), 7.72–7.78 (m, 0.3H), 7.79–7.85 (m, 0.4H), 7.88–7.94 (m, 0.9H).

^13^C NMR (DMSO-*d*_6_) δ [ppm]: 176.97, 176.02, 172.61, 170.64, 168.85, 167.21, 166.73, 158.96, 153.27, 143.09, 131.81, 131.55, 131.31, 129.52, 128.97, 128.82, 128.69, 127.50, 126.76, 123.12, 121.84, 119.05, 115.31, 75.90, 63.24, 62.03, 53.67, 52.19, 50.86, 49.92 50.34, 34.47, 31.33, 29.02, 28.70, 28.59, 27.31, 26.85, 25.92, 25.26, 24.84, 22.07, 21.80, 19.04, 18.87, 14.02.

#### Tetramethylene-1,4-bis(decyldimethylammonium) Tribenuron-methyl, 3,6-Chloro-2-methoxybenzoate (1:1) (**7**), after Decomposition

^1^H NMR (DMSO-*d*_6_) δ [ppm]: 0.86 (t, *J* = 6.8 Hz, 6H), 1.18–1.34 (m, 28H), 1.57–1.84 (m, 8H), 2.21 (s, 2.4H), 2.26 (s, 1.8H), 2.76–2.82 (m, 4.5H), 3.04 (s, 12H), 3.21–3.32 (m, 7H), 3.32–3.42 (m, 4H), 3.74 (s, 2.5H), 3.78–3.82 (m, 5.4H), 3.83–3.87 (m, 3.2H), 7.07 (d, *J* = 8.5 Hz, 1H), 7.21 (d, *J* = 8.5 Hz, 1H), 7.30–7.39 (m, 1.2H), 7.43–7.53 (m, 1.7H), 7.58–7.63 (m, 0.6H), 7.64–7.70 (m, 0.5H), 7.70–7.77 (m, 1H), 7.78–7.84 (m, 0.8H), 7.88–7.93 (m, 0.8H), 7.97–8.02 (m, 0.2H).

^13^C NMR (DMSO-*d*_6_) δ [ppm]: 176.99, 175.98, 170.62, 170.11, 168.85, 167.94, 167.17, 166.93, 165.26, 158.90, 151.21, 143.07, 141.53, 134.79, 131.95, 131.79, 131.60, 131.24, 131.02, 130.78, 129.53, 128.96, 128.89, 128.73, 127.51, 126.85, 126.79, 125.30, 125.14, 122.47, 119.09, 63.23, 62.00, 61.03, 53.68, 52.85, 52.20, 50.87, 49.92, 31.31, 29.04, 28.89, 28.66, 28.61, 27.34, 26.87, 25.80, 25.32, 24.76, 22.08, 21.82, 18.96, 14.01.

#### Dodecylmethylene-1,12-bis(decyldimethylammonium) Tribenuron-methyl, 2,4-Dichlorophenoxyacetate (1:1) (**8**), after Decomposition

^1^H NMR (DMSO-*d*_6_) δ [ppm]: 0.85 (t, *J* = 6.8 Hz, 6H), 1.16–1.38 (m, 44H), 1.55–1.71 (m, 8H), 2.14 (s, 3H), 2.20 (s, 2.1H), 2.25 (s, 1.4H), 2.76–2.82 (m, 3.4H), 2.99 (s, 12H), 3.19–3.25 (m, 8H), 3.28 (s, 1.5H), 3.72 (s, 1.6H), 3.78 (s, 1.5H), 3.83 (s, 2H), 4.19 (s, 2H), 6.68 (d, *J* = 8.7 Hz, 1H), 7.07 (dd, *J*_12_ = 2.6 Hz, *J*_13_ = 8.8 Hz, 1H), 7.13 (d, *J* = 2.9 Hz, 1H), 7.29–7.33 (m, 0.5H), 7.40–7.50 (m, 1.1H), 7.54–7.60 (m, 1.1H), 7.62–7.66 (m, 0.4H), 7.71–7.77 (m, 0.4H), 7.78–7.85 (m, 0.6H), 7.87–7.91 (m, 0.5H).

^13^C NMR (DMSO-*d*_6_) δ [ppm]: 177.03, 175.98, 172.81, 170.63, 170.12, 168.79, 167.76, 167.10, 166.87, 158.85, 156.03, 145.34, 143.15, 134.79, 131.82, 131.47, 130.93, 129.40, 129.26, 128.89, 128.68, 127.83, 126.65, 125.91, 122.64, 122.44, 118.98, 112.86, 67.94, 62.81, 53.65, 52.02, 50.77, 49.89, 31.34, 29.01, 28.89, 28.78, 28.72, 28.64, 28.49, 28.44, 27.28 26.91, 25.75, 25.66, 25.20, 22.13, 21.68, 21.63, 15.96, 13.92.

### NMR Analysis

The structures of the obtained salts were confirmed by analysis of nuclear magnetic resonance spectra (^1^H and ^13^C). The NMR spectra were recorded by a Varian Mercury spectrophotometer operating at 400 MHz for the ^1^H spectrum and at 100 MHz for the ^13^C spectrum. Tetramethylsilane (TMS) was used as an internal standard.

### Solubility

The solubility of the prepared salts was determined according to Vogel’s Textbook of Practical Organic Chemistry.^26^ Ten representative, popular solvents were chosen and ranked in descending order of Snyder polarity index value (water, 9.0; methanol, 6.6; DMSO, 6.5; acetonitrile, 6.2; acetone, 5.1; ethyl acetate, 4.3; 2-propanol, 4.3; chloroform, 4.1; toluene, 2.3; hexane, 0.0). “Complete solubility” applies to compounds that dissolve (0.1 g of IL) in 1 mL of the solvent, while “limited solubility” means that compounds dissolve in 3 mL of the solvent. The “insoluble” term was used to classify compounds that did not dissolve in 3 mL of the solvent. All samples were thermostated at 25 °C.

### Herbicidal Activity

Common lambsquarters (*Chenopodium album* L.) and oilseed rape (*Brassica napus* L.) cornflower (*Centaurea cyanus* L.) were grown in the greenhouse in 0.5 L plastic pots filled with commercial peat-based potting material. The greenhouse was maintained at 20 ± 2 °C at an air humidity 60–80% and photoperiod of 16/8 day/night hours. The seedlings were thinned to five uniform plants per pot within 10 days after emergence. Plants were watered and fertilized as needed for healthy growth. All tested HILs were applied in doses corresponding to 15 g ha^–1^ tribenuron-methyl. The content of the second herbicide anion in the tested ionic liquids, such as 2,4-D, MCPA, MCPP, 2,4,5-T, 4-CPA, or dicamba, ranged from 16.58 to 23.66%; therefore, the dose of these herbicides was from 7.057 to 9.678 g ha^–1^ depending on the type of anion. Lumer 50 WG (tribenuron-methyl 50%, ADAMA, Poland) was used at 30 g ha^–1^ as a reference herbicide. Treatments were applied using a moving nozzle sprayer delivering 200 L ha^–1^ of spray solution from a flat-fan TeeJet 1102 nozzle (TeeJet Technologies, Wheaton, IL, USA) at 0.2 MPa operating pressure. The plants were treated once at the 5–6 leaf stage with a water solution of the tested compounds. Shoot fresh weight was determined 4 weeks after herbicide application using a Sartorius BP 2000 S balance with 0.001 g precision (Sartorius, Göttingen, Germany). Data are expressed as the percent of fresh weight reduction compared to nontreated plants. The experiments were performed twice in completely randomized setups with four replications. Data from individual experiments were combined.

The error margin range represents standard errors of the mean (SEM). The SEM values were calculated according to the equation
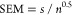
where SEM is the standard error of the mean, *s* is the sample standard deviation, and *n* is the number of samples.

Data were statistically analyzed using one-way ANOVA with a random series effect. Tukey’s multiple post hoc test (α = 0.05) was used to compare treatments.

Moreover, in a separate experiment, the effect of selected ionic liquids on the biotype of cornflower resistant to acetolactate synthase (ALS) inhibitors was tested. The resistance trait in the cornflower population was confirmed in earlier greenhouse tests. The ED_50_ was more than 480 g ha^–1^ of tribenuron-methyl. Plants were prepared, and treatments were applied in the same manner as described above.

Each of the two series of experiments was conducted in a completely randomized design. For this experiment, two-way ANOVA preceding Tukey’s multiple post hoc test (α = 0.05) was carried out (α = 0.05). The two-way ANOVA analyzed the effect of the independent variables (factor A, treatment; factor B, kind of biotype) on the efficacy. The program XLStat Premium was used for the calculations.

## Results

**Scheme 1:**
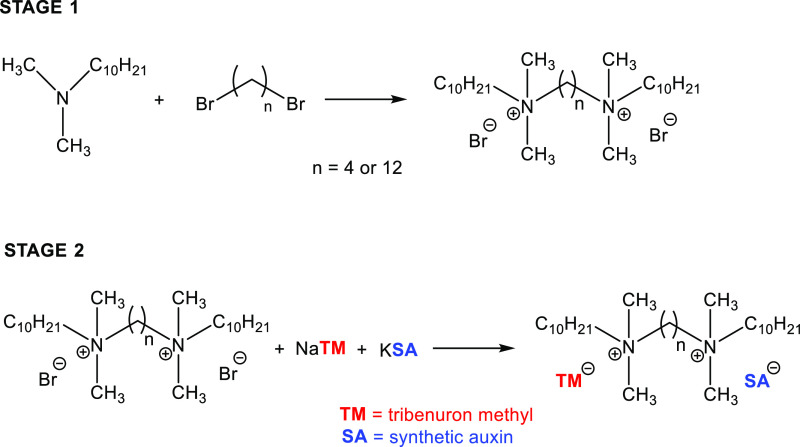
Synthesis of Dicationic Bromides and New HILs

In this study, we demonstrate a group of 14 new HILs that had not been previously described in the literature. The products contained two dicationic cations in their structure, tetramethylene-1,4-bis(decyldimethylammonium) and dodecylmethylene-1,12-bis(decyldimethylammonium), and two different anions, one from the group of synthetic auxins and the second being sulfonylureas. The process was conducted in two stages. In the first stage, precursors of HILs—tetramethylene-1,4-bis(decyldimethylammonium) chloride and dodecylmethylene-1,12-bis(decyldimethylammonium) chloride—were obtained by quaternizing decyldimethylamine with 1,4-dibromobutane or 1,12-dibromododecane according to Scheme 1.

Quaternization reactions were conducted in acetonitrile at 82 °C for 24 h. Subsequently, the solvent was evaporated, and ethyl acetate was added. In effect, the products precipitated in the form of white petals. After filtering and washing, the sediments were thoroughly dried. The yield of this step was equal to 90% for the compound with the C_4_ linker and 92% for the dibromide with the C_12_ linker.

**Scheme 2:**
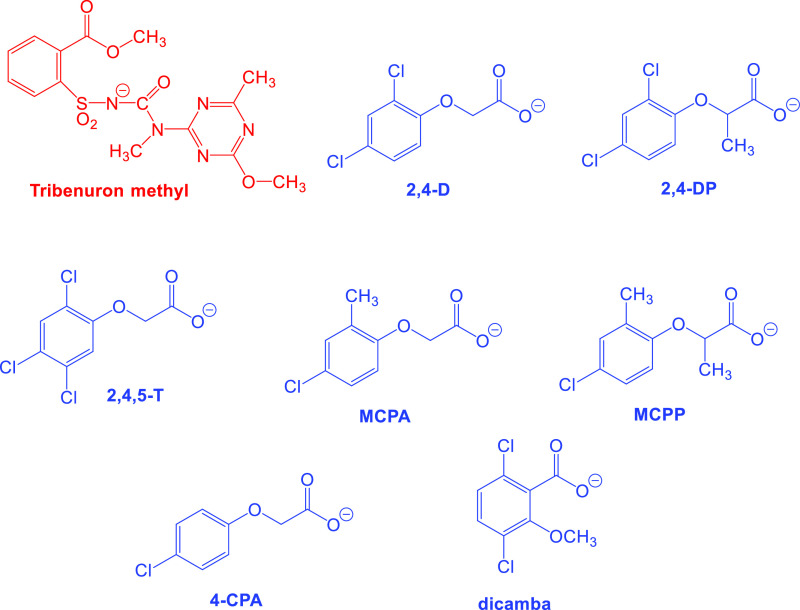
Structures of Herbicidal Anions Present in the Obtained Products

The next stage of the syntheses (Scheme 1) was based on the exchange of bromide anions in the precursors for the two selected, different herbicides, one of which was a tribenuron-methyl (sulfonylurea, TBM) in all cases, while the second was an herbicide from the group of synthesized auxins, such as 3,6-dichloro-2-methoxybenzoate (dicamba) or phenoxy acid from the groups 2,4-dichlorophenoxyacetate (2,4-D), 2-(2,4-dichlorophenoxy)propionate, (2,4-DP), 2,4,5-trichlorophenoxyacetate (2,4,5-T), 4-chloro-2-methylphenoxyacetate (MCPA), 2-(4-chloro-2-methylphenoxy)propionate (MCPP), or 4-chlorophenoxyacetate (4-CPA), which are presented in Scheme 2. Thus, we have included a wide spectrum of well-known herbicidal anions in the designed ILs. It should be noted that although 2,4,5-T acid has been declassified as an herbicide, we also obtained compounds derived from it for comparative purposes.

Among the organic solvents tested, methanol turned out to be the best solvent for the anion exchange reaction, which enabled the simple separation of precipitated potassium bromide (or sodium bromide) and obtained high yields (over 90%) in less than 1 h of reaction time. The products were further purified by dissolving the residue in acetone and then filtering off the insoluble impurities. Finally, after acetone evaporation, the obtained HILs were thoroughly dried in a vacuum oven. The purity of the obtained products was determined using the two-phase titration technique according to the PN-EN ISO 2871-2:2010 standard and ranged from 98 to 99%. Table 1 lists the synthesized HILs containing the dicationic cation and two different herbicidal anions.

**Table 1:** Synthesized ILs Containing the Dicationic Cation and Two Different Herbicidal Anions

		anion			
ILs	*n*	TM	SA	yield (%)	purity (%)
**1**	4	tribenuron methyl	2,4-D	97	98
**2**			2,4-DP	94	99
**3**			2,4,5-T	95	98
**4**			MCPA	97	98
**5**			MCPP	96	99
**6**			4-CPA	99	98
**7**			dicamba	95	99
**8**	12		2,4-D	95	99
**9**			2,4-DP	95	98
**10**			2,4,5-T	92	98
**11**			MCPA	96	99
**12**			MCPP	99	98
**13**			4-CPA	96	99
**14**			dicamba	97	99

The structures of the products obtained were confirmed by proton and carbon nuclear magnetic resonance (^1^H and ^13^C NMR). For example, the proton spectrum of product **9** contained signals in the range of 6.8–8.2 ppm from both herbicidal anions (TBM and 2,4-DP) and a signal at 0.9 ppm from methyl groups in the alkyl chains present in the dicationic cation. This showed the presence of all three assumed ions that are incorporated into the structure of this IL.

**Figure 1:**
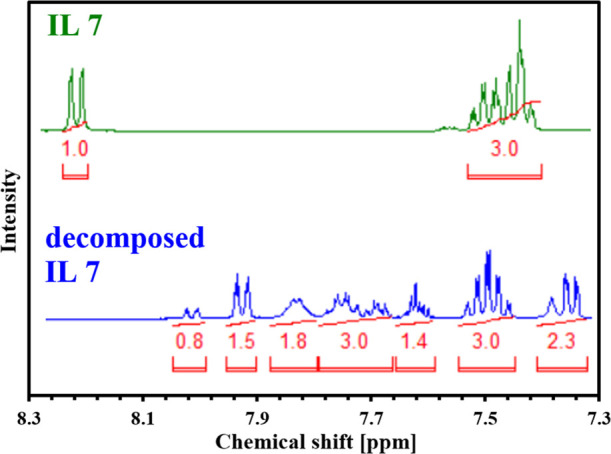
^1^H NMR spectra of IL **7** after synthesis (green) and after decomposition of sulfonylurea anion (blue).

Unexpectedly, all the obtained compounds turned out to be unstable during storage, and depending on the compound, after 2–8 weeks after the synthesis, there was a noticeable change in their appearance. This is in contradiction to other findings regarding HILs containing other sulfonylurea-based herbicides such as metsulfuron-methyl,^27^ iodosulfuron-methyl,^28,29^ or nicosulfuron,^30^ wherein these compounds were stable even after months of storage. Tribenuron-methyl is a direct analogue of metsulfuron-methyl and iodosulfuron-methyl; however, it contains an additional methyl group within the sulfonylurea bridge, which is known to be extremely susceptible to degradation. Hence, we may assume that the presence of this extra methyl group influences the distribution of charge in the sulfonylurea group and facilitates its decomposition. This phenomenon is illustrated in Figure 1, which demonstrates the differences in ^1^H NMR spectra between IL **7** after synthesis and 4 weeks of storage. We can notice that the pure products possess only two signals from hydrogens present in the aromatic ring at approx. 7.5 ppm (multiplet) and 8.2 ppm (doublet). After decomposition, the spectrum contained multiple other signals that occurred mainly in the region between 7.3–8.0 ppm. The differences in the NMR spectra provided in the Figure 1 indicate that the structure of the functional group directly attached to the aromatic ring has been altered. As a result, the most significant changes in the position of the signals were noted for the protons present in the atomic ring (which appear in a region of 6–8 ppm). This means that the presence of an additional methyl group in the sulfonylurea bridge caused a reduction in its stability and, in consequence, faster decomposition to the appropriate sulfonoamide and aminotriazine. The confirmation of proposed degradation pathway requires further experiments; however, the collected data are in agreement with recent reports describing other sulfonylureas.^29^

### Solubility

The solubility of the active ingredient in the herbicide formulation is an important characteristic that determines the choice of work solution composition. To determine the affinity of the obtained systems, we performed a series of tests using water and nine organic solvents with a wide spectrum of polarities. The results of the solubility experiment are presented in Table 2. Due to the presence of the amphiphilic bis(ammonium) cation with an extended chemical structure, most of the ILs tested dissolved readily or to a limited extent in 8 of the 10 solvents tested regardless of the phenoxy acid anion. It should be noted that two dicamba-based ILs, **7** and **14**, were exceptions and, unlike the others, showed poor solubility in acetonitrile. The limited solubility of dicamba-containing ILs in this solvent is in line with previous works.^12,31^ Moreover, none of the ILs dissolved noticeably in hexane or ethyl acetate, and in another solvent of low polarity, toluene, the obtained compounds dissolved only to a limited extent. Despite the presence of the two hydrophobic anions, ILs **1**–**14** were characterized by a noticeable affinity for water, the most commonly used solvent in spray solutions.

**Table 2:** Solubility of Synthesized Dicationic HILs (**1**–**14**) at 25 °C

HIL	water (9.0^a^)	methanol (6.6)	DMSO (6.5)	acetonitrile (6.2)	acetone (5.1)	isopropanol (4.3)	ethyl acetate (4.3)	chloroform (4.1)	toluene (2.3)	hexane (0.0)
**1**	+	+	+	+	+	+	–	+	±	–
**2**	+	+	+	+	+	+	–	+	±	–
**3**	+	+	+	+	+	+	–	+	±	–
**4**	+	+	+	+	+	+	–	+	±	–
**5**	+	+	+	+	+	+	–	+	±	–
**6**	+	+	+	+	+	+	–	+	±	–
**7**	+	+	+	–	+	+	–	+	±	–
**8**	±	+	+	+	+	+	–	+	±	–
**9**	±	+	+	+	+	+	–	+	±	–
**10**	±	+	+	+	+	+	–	+	±	–
**11**	±	+	+	+	+	+	–	+	±	–
**12**	±	+	+	+	+	+	–	+	±	–
**13**	±	+	+	+	+	+	–	+	±	–
**14**	+	+	+	–	+	+	–	+	±	–

a Snyder polarity index; +, soluble; ±, limited solubility; −, not soluble.

Moreover, it should be emphasized that the solubility in water depended on the length of the alkylene linker in the cation structure. HILs **1**–**7** containing a butylene linker dissolved in water significantly easier than their analogues with a dodecylene linker (ILs **8**–**14**), which showed limited solubility. These results confirmed previous findings regarding dicationic ILs with hydrophobic anions^22^ as well as the fact that it is possible to select favorable properties such as an appropriate solubility level in the desired solvent during the stage of designing the structures of new ILs.^9,32^

### Herbicidal Activity

**Figure 2:**
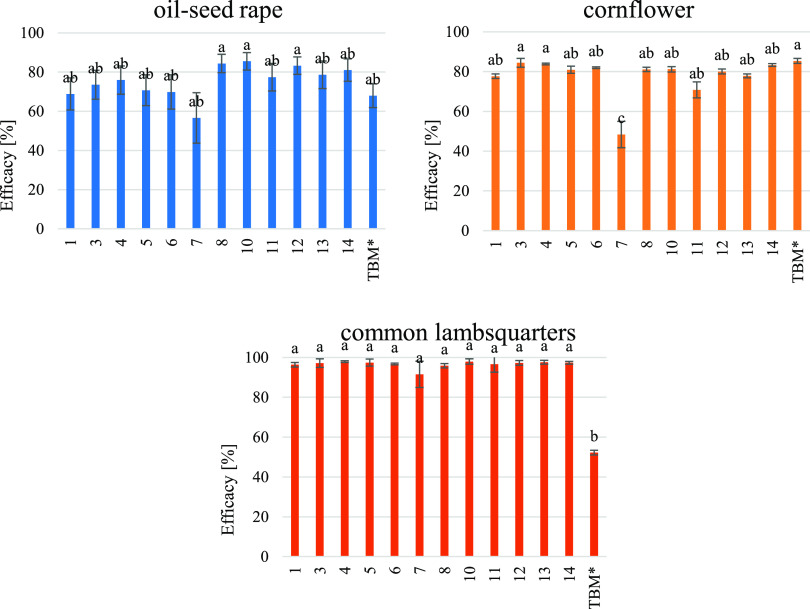
Fresh weight reduction of target plants treated with HILs (**1**, **3**–**8**, and **10**–**14**) and commercial herbicide (TBM*).

As shown in Figure 2, all tested new ILs demonstrated herbicidal activity. The effectiveness of weed control depended primarily on the plant species as well as the structure of ILs. Compounds with cations having short alkyl chains (C_4_) were less effective than those with longer alkyl chains (C_12_). This relationship was evident in the control of oilseed of rape plants. The efficacy of ILs with short alkyl chains ranged from 39 to 76%, while ILs with longer alkyl chains controlled oilseed of rape plants at 77–85%. We previously presented the influence of alkyl chain length on the biological activity of herbicidal ionic liquids.^33^ Tested HILs showed excellent efficacy against common lambsquarter plants. The fresh weight reduction was over 91%.

The herbicidal activity of the HILs with an alkyl chain containing 12 carbon atoms on the rapeseed plants was 10–17% higher than that of the reference herbicide. No significant differences were found between the efficacy of HILs and the reference herbicide. In the case of common lambsquarters, the effectiveness was over 40% higher compared to the reference product, which was a statistically significant difference. Different results were obtained in the control of cornflower. No significant differences were found between the efficacy of HILs and the reference herbicide except for compound **7**, which showed significantly lower effectiveness compared to other treatments.

#### Influence of Selected Ionic Liquids on Cornflower Resistance to ALS Inhibitors

**Figure 3:**
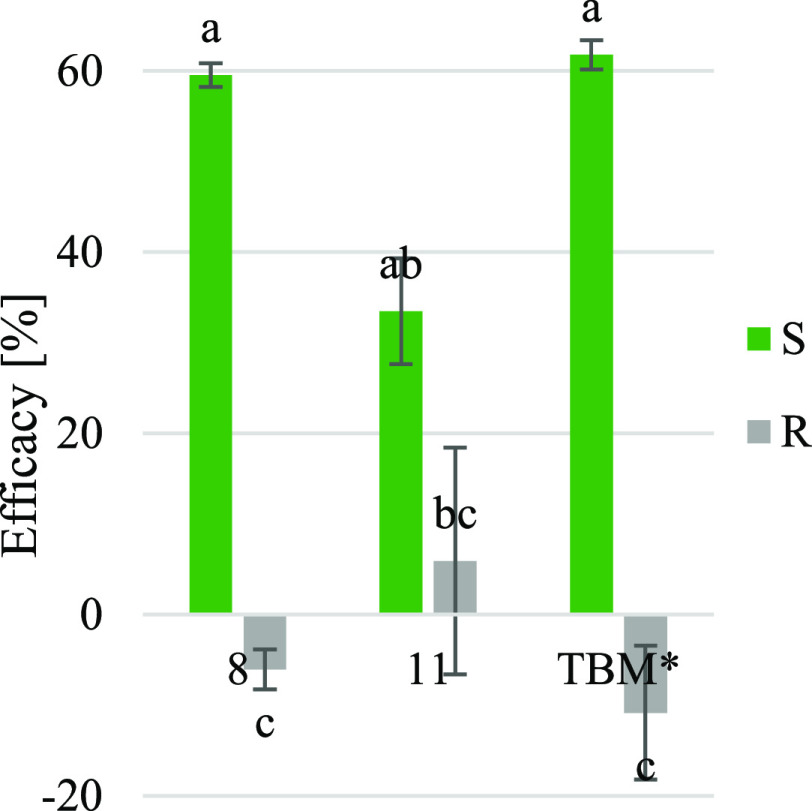
The influence of the tested HILs and reference herbicide on the cornflower biotypes resistant (R) and sensitive (S) to ALS inhibitors.

HILs **8** and **11** were used to control the cornflower biotype resistant to tribenuron-methyl. The resistance of this biotype to tribenuron-methyl was at a very high level because the effective dose (ED_50_) was 480 g ha^–1^, while the recommended dose was 15 g ha^–1^. As shown in Figure 3, all tested compounds showed herbicidal activity on cornflower sensitive (S) to tribenuron-methyl, while reference herbicide and **8** did not control biotype resistant (R) to this herbicide and even increased plant biomass, while **11** caused slight symptoms of plant damage.

Although the differences in the efficacy of HILs on the R biotype were statistically not significant, the obtained results indicate that ionic liquids containing an anion from the phenoxy acid group may limit the development of cornflower resistance to ALS. However, increasing the content of such anions in the structure of compounds would likely improve the effectiveness.
